# Liposomes for Targeted Delivery of Active Agents against Neurodegenerative Diseases (Alzheimer's Disease and Parkinson's Disease)

**DOI:** 10.1155/2011/469679

**Published:** 2011-12-13

**Authors:** Carlos Spuch, Carmen Navarro

**Affiliations:** Department of Pathology and Neuropathology, University Hospital of Vigo (CHUVI), Hospital of Meixoeiro, Meixoeiro s/n, 36215 Vigo, Spain

## Abstract

Neurodegenerative diseases, such as Alzheimer's disease and Parkinson's disease represent a huge unmet medical need. The prevalence of both diseases is increasing, but the efficacy of treatment is still very limited due to various factors including the blood brain barrier (BBB). Drug delivery to the brain remains the major challenge for the treatment of all neurodegenerative diseases because of the numerous protective barriers surrounding the central nervous system. New therapeutic drugs that cross the BBB are critically needed for treatment of many brain diseases. One of the significant factors on neurotherapeutics is the constraint of the blood brain barrier and the drug release kinetics that cause peripheral serious side effects. Contrary to common belief, neurodegenerative and neurological diseases may be multisystemic in nature, and this presents numerous difficulties for their potential treatment. Overall, the aim of this paper is to summarize the last findings and news related to liposome technology in the treatment of neurodegenerative diseases and demonstrate the potential of this technology for the development of novel therapeutics and the possible applications of liposomes in the two most widespread neurodegenerative diseases, Alzheimer's disease and Parkinson's disease.

## 1. Introduction

Each year, over 10 million people globally suffer from neurodegenerative diseases. This figure is expected to grow by 20% over the next decade as the aging population increases and lives longer. Neurodegenerative diseases are the fourth leading cause of death in the developed world after heart diseases, cancer, and stroke [1]. There are millions of sufferers worldwide, and the start of the disease can occur at any age, but it is more common among the elderly. Many similarities appear that relate these diseases to each other on a subcellular level [2]. Discovering these similarities offers hope for therapeutic advances that could ameliorate many diseases simultaneously.

The most common neurodegenerative diseases are Alzheimer's disease, Parkinson's disease, Lewy body dementia, frontotemporal dementia, amyotrophic lateral sclerosis, Huntington's disease, and prion diseases [3]. The most widely recognized are Alzheimer's disease and Parkinson's disease, which are among the principal debilitating conditions of the current century. Approximately 24 million people worldwide suffer from dementia, 60% of cases being due to Alzheimer's disease, which occurs in 1% of individuals aged 50 to 70 and dramatically increases to 50% for those over 70 years [4]. Dramatically, these numbers are estimated to increase to 15 million in the next 40 years [5].

Alzheimer's disease is typified clinically by learning and memory impairment and pathologically by gross cerebral atrophy, indicative of neuronal loss, with numerous extracellular neuritic amyloid plaques and intracellular neurofibrillary tangles found predominantly in the frontal and temporal lobes, including the hippocampus [6]. The mechanisms underlying Alzheimer's disease are not completely clear yet, and there is still no cure. However, in recent years, several approaches aimed at inhibiting disease progression have advanced to clinical trials. Among these, strategies targeting the production and clearance of the amyloid-beta peptide are the most advanced [7]. The predominant accumulation and initial peptide deposited in the brain parenchyma is a highly fibrillogenic amyloid-beta 1-42 [8]. Oligomers appearing before plaque deposition in an early stage of Alzheimer's disease pathology have been indicated as the most toxic amyloid-beta species [9]. Targeting amyloid-beta 1-42 in all its aggregation forms has been suggested for therapeutic and diagnostic purposes [10, 11]. Moreover, it has been recently demonstrated that brain and blood amyloid-beta are in equilibrium through the BBB, and sequestration of amyloid-beta in the blood may shift this equilibrium, drawing out the excess from the brain [12–14].

Parkinson's disease is a chronic and progressive neurological disease, the symptoms of which include tremors, stiffness and slow or hesitant speech. While the disease is most commonly associated with older people, it is thought that around one in ten people are diagnosed before the age of 50. There are now almost 1.2 million people suffering from Parkinson's disease in Europe and over 1 million in US; however, medication only provides patients with temporary symptomatic relief, while access to care and treatment differs widely depending on where patients live [15]. Parkinson's disease is characterized by massive depletion of striatal dopamine as a result of degeneration of dopaminergic neurons in the substantia nigra pars compacta. Beside the lack of dopamine at the cellular level the formation of Lewy bodies in the substantia nigra, which are cytoplasmic inclusions composed of fibrils, ubiquitin, and alpha-synuclein may appear [16, 17].

Pharmaceutical agents that are used to treat neurodegenerative diseases are usually administered orally, such as donepezil, memantine, rivastigmine, galantamine and tacrine for Alzheimer's disease [18], or levodopa, entacapone, pramipexole, ropinrole, benserazide, carbidopa, tolcapone, entacapone, selegiline, rasagiline, and safinamide for Parkinson's disease [19]. However, most of the ingested drugs do not reach the brain in a fully way and are, instead, metabolized totally or partially by the liver. This inefficient utilization of drug may require ingestions of higher drug concentrations that can produce toxic effects in the heart, liver, or kidney. Also, many therapeutic agents are poorly soluble or insoluble in aqueous solutions. These drugs provide challenges to deliver them orally or parentally, however, these compounds can have significant benefits when formulated through other technologies such as liposomes.

Drug delivery to the brain remains the major challenge for the treatment of all neurodegenerative diseases because of the numerous protective barriers surrounding the central nervous system. Various strategies have been developed to deliver drugs into the brain that would not otherwise be able to cross the BBB. Commonly, although quite undesirable, an intraventricular catheter is surgically implanted to deliver a drug directly into the brain. New therapeutic drugs that cross the BBB are critically needed for treatment of many brain diseases. One of the significant factors on neuro-therapeutics is the constraint of the BBB and the drug release kinetics that cause peripheral serious side effects. Contrary to common belief, neurodegenerative and neurological diseases may be multisystemic in nature, and this presents numerous difficulties for their potential treatment. In recent years, gene therapy has evolved as a new treatment for brain diseases, especially for neurodegenerative diseases, where genetically engineered cells can be used to deliver specific growth factors to target cells. However, clinical troubles have been limiting this technique due to insufficient gene transfer, lack of prolonged gene expression, or immunorejection of producer cells. One promising technology is the development of new biomaterial components with the capacity of enveloping genetically engineered cells producing and distributing the drug therapy, and, at the same time, to be isolated from immune system. This technology includes, among others, the liposomes representing a potential delivery system for specific proteins and growth factors to brain damage, where different producer cells may be isolated from the microenvironmental factors [20].

**Figure 1:**
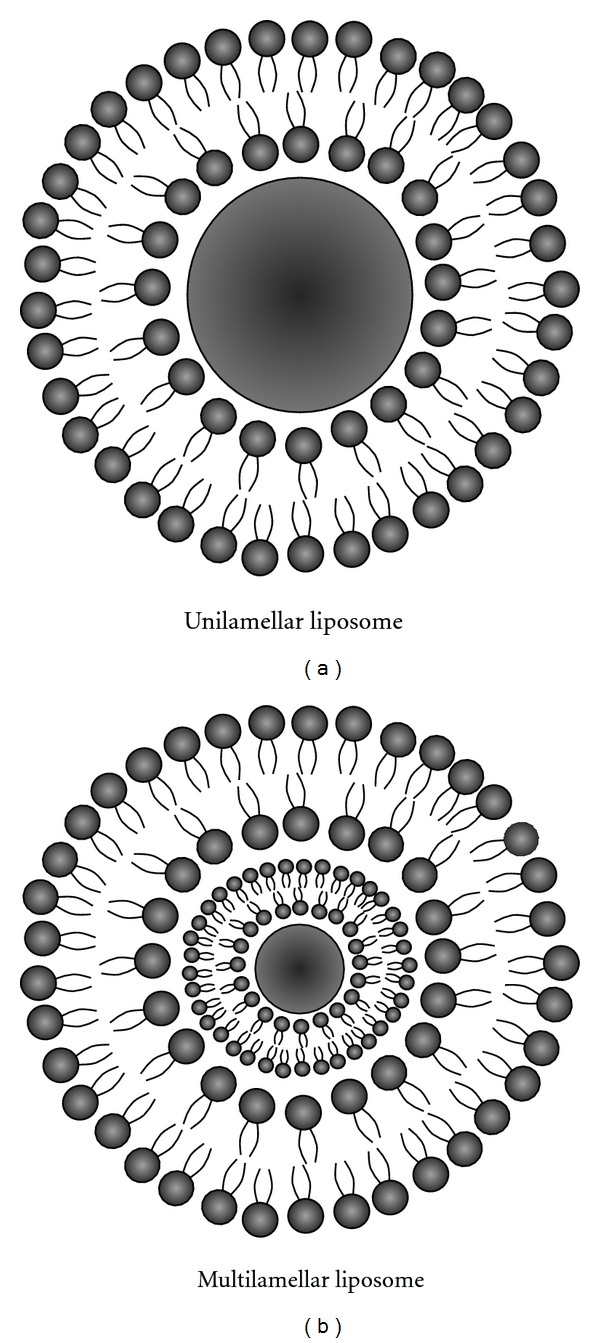
Schematic representation of the basic structure of unilamellar liposomes (a) and multilamellar liposomes (b). The aqueous core of the liposome, loaded with the drug, is surrounded by a phospholipid bilayer.

Liposomes are spherical vesicle structures composed of a uni- or multilamellar lipid bilayer surrounding internal aqueous compartments and a relatively impermeable outer lipophilic phospholipid bilayer (Figure 1). Liposomes have gained considerable attention as drug delivery carriers because they are biocompatible, nontoxic, can deliver both hydrophilic and lipophilic drug molecules, protect their cargo from degradation by plasma enzymes, and transport their load across biological membranes and the BBB [21, 22].

Overall, this paper provides an overview of progress in liposome technology, summarizing the last patents and news related to liposomes, demonstrating the potential of this technology for the development of novel neurotherapeutics and its applications in Alzheimer's disease and Parkinson's disease.

## 2. Blood-Brain Barrier

The central nervous system (CNS) barriers are composed by BBB and blood-cerebrospinal fluid barrier (B-CSFB). The BBB and B-CSFB are a highly specialized brain endothelial and epithelial structure of the fully differentiated neurovascular system. These barriers separate components of the circulating blood from neurons. Moreover, the BBB and B-CSFB maintain the chemical composition of the neuronal “milieu,” which is required for the proper functioning of neuronal circuits, synaptic transmission, synaptic remodelling, angiogenesis, and neurogenesis in the adult brain.

Three barrier layers regulate molecular exchange at the interfaces between blood and the neural tissue or its fluid spaces: the BBB formed by the cerebrovascular endothelial cells between blood and brain interstitial fluid, the B-CSFB formed by the choroid plexus epithelium between blood and ventricular CSF, and the third barrier is the arachnoids epithelium between blood and subarachnoid CSF. Since individual neurons are extremely close to the brain capillaries, rarely at a distance greater than 20 *μ*m, of the various CNS barriers, these barriers exert the greatest control over the immediate microenvironment of brain cells [23].

**Figure 2:**
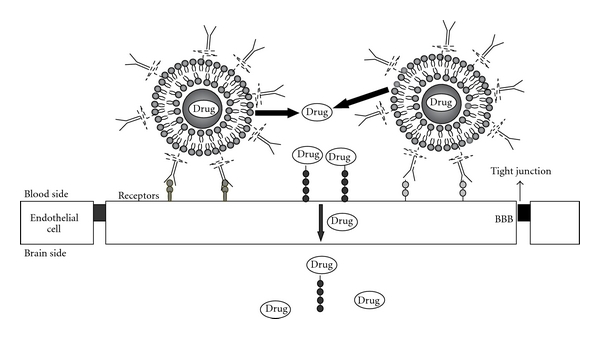
The antibodies of the immunoliposomes recognise the specific receptor of the BBB releasing the drug of the immunoliposomes only close to the targeting cells.

Due to the presence of multiple endogenous transporters, BBB allows a selective entry of nutrients and minerals across it and limits the entry of foreign substances like drugs as well as neuropharmaceutical agents. This makes the CNS treatment ineffective. The conventional drug delivery systems which release the drug into general circulation fail to deliver drugs effectively to brain and are, therefore, not very useful in treating certain diseases that affect CNS including Alzheimer's disease, dementia, Parkinson's disease, mood disorders, AIDS, and viral and bacterial meningitis. Therefore, there is a need to develop and design approaches which specifically target to brain in a better and effective way [24] (Figure 2).

Drug delivery to the brain is hindered by the presence of the CNS barriers. Although the BBB and B-CSFB restrict the passage of many substances, both are actually selectively permeable to nutrients necessary for healthy brain function. In the last decades, despite the advances in drug discovery, there has been little improvement on the prognosis of patients with brain diseases, like cancer, neurodegenerative diseases, or neurological disorders. Often, in clinical trials it has been found that promising agents *in vitro* have little impact on the disease. These disappointing results can be, at least in part, explained by the inability to deliver therapeutic agents to the brain across CNS barriers avoiding various resistance mechanisms to reach the desired targets [25].

## 3. Liposomes

Liposomes have received widespread attention as a carrier system for therapeutically active compounds, due to their unique characteristics such as capability to incorporate hydrophilic and hydrophobic drugs, good compatibility, low toxicity, lack of immune system activation, and targeted delivery of bioactive compounds to the site of actions [26].

Liposomes are spherical vesicles that resemble cells in that they contain an inner hydrophilic core and a relatively impermeable outer lipophilic phospholipid bilayer [22, 27]. The lipid components of liposomes are predominantly phosphatidylcholine derived from egg or soybean lecithin [28]. Liposomes have been shown to provide stable encapsulation for various drugs and offer distinct advantages over unencapsulated agents; thus, liposomes have been proposed for use in a variety of applications in research, industry, and medicine, particularly for the use as carriers of diagnostic and therapeutic compounds (Figure 1).

Liposomes are synthetic lipid spheres composed by fatty acid on polymers with a bilayered membrane structure surrounding an aqueous core that can be used to encapsulate small molecules. Liposomes have the distinct advantages of being both nontoxic and biodegradable because they are composed of naturally occurring substances. Biologically active materials encapsulated within liposomes are protected to a varying extent from immediate dilution or degradation, suggesting drug carrier systems for the transport of drugs and other bioactive capsules to disease-affected organs. The unique ability of liposomes to entrap drugs both in an aqueous and a lipid phase make such delivery systems attractive for hydrophilic and hydrophobic drugs. Lipophilic drugs are generally entrapped almost completely in the lipid bilayers of liposomes, and, since they are poorly water soluble, problems like loss of an entrapped drug on storage are rarely encountered. Hydrophilic drugs may either be entrapped inside the aqueous cores of liposomes or be located in the external water phase. Noteworthy is that the encapsulation percentage of hydrophilic drugs by liposomes depends on the bilayer composition and preparation procedure of the liposomes [29]. Furthermore, such encapsulation has been shown to reduce drug toxicity while retaining or improving the therapeutic efficacy.

Liposomes can be made from several different types of lipids; however, phospholipids are most commonly used to generate liposomes as drug carriers. Although liposome formation is spontaneous when a lipid film is mixed with an aqueous solution, it can also be expedited by applying force in the form of shaking by using a homogenizer, sonicator, or an extrusion apparatus [30].

Several other additives may be added to liposomes in order to modify their structure and properties. For instance, either cholesterol or sphingomyelin may be added to the liposomal mixture in order to help stabilize the liposomal structure and to prevent the leakage of the liposomal inner cargo [22]. Further, liposomes are prepared from hydrogenated egg phosphatidylcholine or egg phosphatidylcholine, cholesterol, and dicetyl phosphate, and their mean vesicle sizes were adjusted to about 50 and 100 nm.

Conventional liposome formulation is mainly comprised of natural phospholipids and lipids such as 1,2-distearoryl-sn-glycero-3-phosphatidyl choline (DSPC), sphingomyelin, egg phosphatidylcholines and monosialoganglioside. Since this formulation is made up of phospholipids only, liposomal formulations have encountered many challenges, one of the ones being the instability in plasma [31]. Several attempts to overcome these challenges have been made, specifically in the manipulation of the lipid membrane. One of these attempts focused on the manipulation of cholesterol. Addition of cholesterol to conventional formulations reduces rapid release of the encapsulated bioactive compound into the plasma [32] or 1,2-dioleoyl-sn-glycero-3-phosphoethanolamine (DOPE) increases the stability [33].

Although there are many classifications, depending on the method of preparation, there are described several different types of liposome vesicles.

1. Multilamellar vesicles (MLVs): spontaneous formation of liposomes and gentle shaking produce big MLVs. This type of liposomes is with multiple concentric lipid layers, with up to fourteen layers, each separated by an aqueous solution [34]. MLVs tend to be present as a heterogeneous mixture, with vesicle sizes ranging from 500 to 5000 nm.
2. Small unilamellar vesicles (SUVs): homogenization of MLV can then result in either SUV or large unilamellar vesicles (LUVs). SUVs are liposomes whose structure contains only one lipid layer and whose average diameter ranges from 25 to 100 nm [21, 28].
3. Large unilamellar vesicles (LUVs): this type of liposomes contains a single lipid layer, and its diameter can range from 200 to 800 nm.

The drug retained and that which leaked were separated from plasma by gel filtration. On the assumption that lipid content does not change, the drug released from each liposome preparation was estimated by a latency percentage calculated from the drug/lipid concentration ratio of the liposome preparation. Polyethylene glycol has also been added to the surface of liposomes in order to prevent liposomal aggregation in solution, to decrease liposomal uptake by the reticuloendothelial system, and to increase the half-life of the liposomal formulation. These types of sterically stabilized liposomes are called stealth liposomes [35, 36].

**Figure 3:**
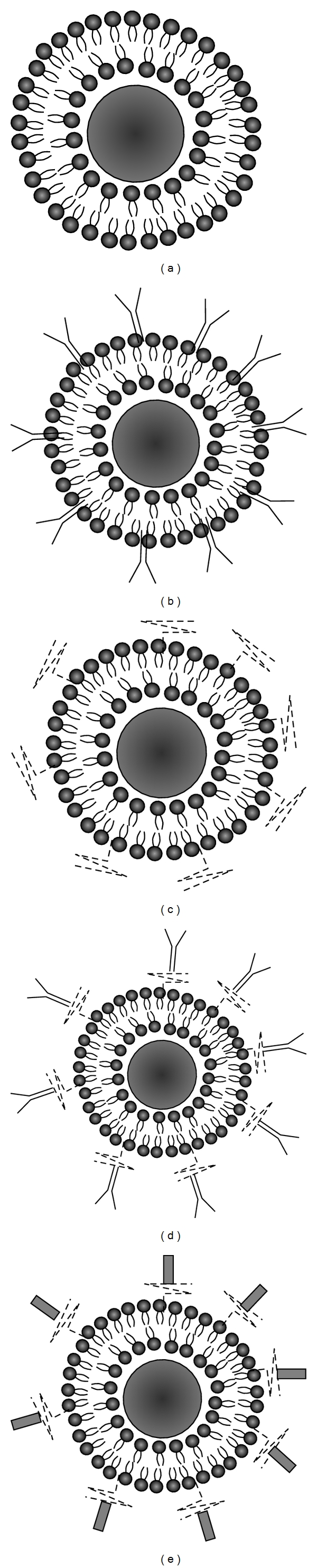
Schematic representation of different types of liposomes. (a) Conventional liposome, (b) conventional liposome tagged directly with antibodies, (c) stealth liposome coated with a polymeric conjugated, (d) liposome coated with a polymeric conjugated tagged with antibodies, (e) liposome coated with a specific ligand.

Stealth liposome technology is one of the most often used liposome-based systems for delivery of active molecules. This strategy was achieved simply by modifying the surface of the liposome membrane, a process that was achieved by engineering hydrophilic polymer conjugates [37]. The employed hydrophilic polymers were natural or synthetic polymers such as polyethylene glycol (PEG), chitosan, silk-fibroin, and polyvinyl alcohol (PVA). Although the majority of hydrophilic polymers conjugate high biocompatibility, nontoxicity, low immunogenicity, and antigenicity, PEG remains the most widely used polymer conjugate (Figure 3).

 The only shortcoming of liposomes involves their difficulty in bypassing certain capillary cells in several organs. In theory, an encapsulated active drug in a liposomal system may be released through three possible mechanisms: passive diffusion, vesicle erosion, and vesicle retention, diffusion, erosion, and retention in the circulation. The liposomes extend then time that medication remains in the blood stream, prolonging therapeutic actions and reducing toxic side effects. Larger size or multilamellar liposomes with a size range of 500–5000 nm were the first to be eliminated from the systemic circulation due to phagocytosis [38]. Their problems, however, are being rectified through modifications of the size and composition of the lipid components.

### 3.1. Delivery Strategies of Liposomes

Although liposomes contain an outer lipophilic membrane that increases their permeability across membranes, some biological barriers such as the BBB remain impenetrable. Also, the charge of the outer membrane affects the distribution and stability of the liposome. Negatively charged liposomes were believed to be more rapidly removed from circulation than neutral or positively charged liposomes; later studies have indicated that the type of negatively charged lipid affects the rate of liposome uptake by the reticule-endothelial system. For example, liposomes containing negatively charged lipids that are not sterically shielded (phosphatidylserine, phosphatidic acid, and phosphatidylglycerol) are cleared more rapidly than neutral liposomes of similar composition. However, liposomes containing sterically shielded lipids (ganglioside-GM_1_ and phosphatidylinositol) are cleared even more slowly than neutral liposomes [35].

Consequently, scientists have attempted to modify the liposomal structure in order to improve liposomal penetration across biological membranes and into their target organs. Targeted liposome-based system was suggested after conventional stealth liposomes failed to evade uptake of active molecules by sensitive normal cells or nonspecific targets [39]. In addition, a targeted ligand can further increase the rate of liposomal drug accumulation in the ideal tissue or cells via overexpressed receptors, antigen and unregulated selectin [40, 41]. Peptides, proteins, and antibodies have been mostly studied as a ligand for directing drug-loaded liposomes into sites of action, due to their molecular structures, which are essentially composed by known amino acid sequences (Figure 1).

The main advantage of these structures is the relatively large quantities of drug that can be incorporated into one compartment. However, liposome structures present various problems related to the administration pathway. Orally administration is difficult because the low pH of the stomach and the presence of bile salts tend to destabilize the liposome complex. Liposomes are very sensitive to pH, light, magnetism, temperature, and ultrasonic waves besides, liposomes are highly susceptible to destruction via uptake by the reticule-endothelial system of the macrophages.

A way of protecting liposomes was studied and patented and consisted in increasing stable bilayers and regulating the release profile of the liposome [36, 40]. Although liposomes contain an outer lipophilic membrane that increases their permeability across membranes, some biological barriers such as the BBB remain impenetrable. The development of a suitable liposomal carrier to encapsulate active compounds is very promising. These liposomes, also named targeted liposomes, are stable enough to be carried out to the brain across the BBB, with the appropriate surface characteristics for an effective targeting and for an active membrane transport. The main goal is the capacity to release growth factors, peptides, proteins, or hormones in a precise location, hold the concentration in a physiological range into the brain, and keep it isolated from immune system attack.

### 3.2. Other Types of Liposomes

#### 3.2.1. Immunoliposomes

Immunoliposomes are a promising variant of liposome technique based on an antibody-conjugated liposomes. Liposomes can carry drugs conjugated with monoclonal antibodies and may be directed against target cells (Figure 2). Although this technique is still in its infancy, significant advances have been made. Immunoliposomes have been successfully used *in vivo* to achieve targeted delivery of tumour-suppressing genes into tumours, using an antibody fragment against the human transferrin receptor. Tissue-specific gene delivery using immunoliposomes has also been achieved in brain [41]. Chimeric or humanized monoclonal antibodies can reduce the host response against the therapeutic antibody [42, 43]. The main problem to solve with these antibodies is to reduce the antigenicity of the immunoliposomes; thus, a possibility is to remove the Fc portion of the IgG antibody reducing antigenicity and increasing the therapeutic efficacy. In addition, cellular internalization of antibodies increases efficacy of drug delivery, presumably by inducing target cells to endocytose immunoliposomes. This is the case with the HER2-targeted immunoliposomes in tumours cells, which distribute within solid tumours and not simply in the extracellular space surrounding the tumour blood vessels.

 As an attempt to achieve active targeting using high-affinity binding of antibody to the target, immunoliposome, a liposome with antibody attached to its surface, was developed. Ordinary liposome conjugated by antibody insufficiently avoids the reticule-endothelial system, so a PEG-modified liposome is necessary. Two types of approach were considered; the antibody is conjugated directly to phospholipid or else to an end of the PEG chain. Experimental results indicated that binding to an end of the PEG chain is essential to preserve antigen recognition capacity. Antibody-conjugated liposome is also called pendant-type immunoliposome because of its shape. Pendant-type immunoliposome is expected to play an important role in active targeting, since it has long retention due to PEG and antigen recognition capacity thanks to antibody conjugation. But in the case of the IgG antibody, macrophages recognize it and uptake in the liver increases, for macrophages having Fc receptors. In order to solve this, an immunoliposome using Fab fragment that lacks Fc region was prepared to have longer retention after intravenous administration than IgG-PEG-liposome [44]. The pattern of Fab-PEG-liposome disappearance in blood was the same as PEG-liposome, and it had two stages of disappearance, namely, an initial, fast disappearance due to phagocytosis by macrophages and a late, slow disappearance. Since longer retention was achieved by Fab-PEG-liposome, it was shown to avoid the initial uptake due to phagocytosis.

#### 3.2.2. Virosomes

New generation types of liposomes have been developed to increase bioactive molecule delivery to the cytoplasm by escape endosome [45]. New approaches that employ liposomes as pharmaceutical carriers are virosomes. A virosome is another type of liposome formulation; it comprises noncovalent coupling of a liposome and a fusogenic viral envelope. Virosomes have been constructed by combining viral components with nonviral vectors or by using pseudovirions without viral genome replication. Viruses, such as influenza virus, HVJ (hemagglutinating virus of Japan; Sendai virus), and hepatitis B virus, have been used in the construction of virosomes. The HVJ-derived vector is particularly promising due to its highly efficient delivery of DNA, siRNA, proteins, and anticancer drugs. Furthermore, the HVJ envelope vector has intrinsic anti-tumour activities including the activation of multiple antitumour immunities and the induction of cancer-selective apoptosis [46]. During the last 10 years, active vaccination with amyloid peptides shows promise for the treatment and prevention of Alzheimer's disease. Several studies in transgenic mouse models of Alzheimer's disease have revealed the potency of vaccination to prevent or even clear amyloid plaques from mouse brain. Several years ago, Zurbriggen et al. described an active vaccination method based on amyloid-beta (1-16) presented on the surface of virosomes, which triggered a dramatic decrease in both amyloid-beta (1-40) and amyloid-beta (1-42) in a double transgenic mouse model of Alzheimer's disease. These uses of virosomes are a promising antigen carrier system against the neuropathology of Alzheimer's disease [47].

#### 3.2.3. Gene-Based Liposomes

**Figure 4:**
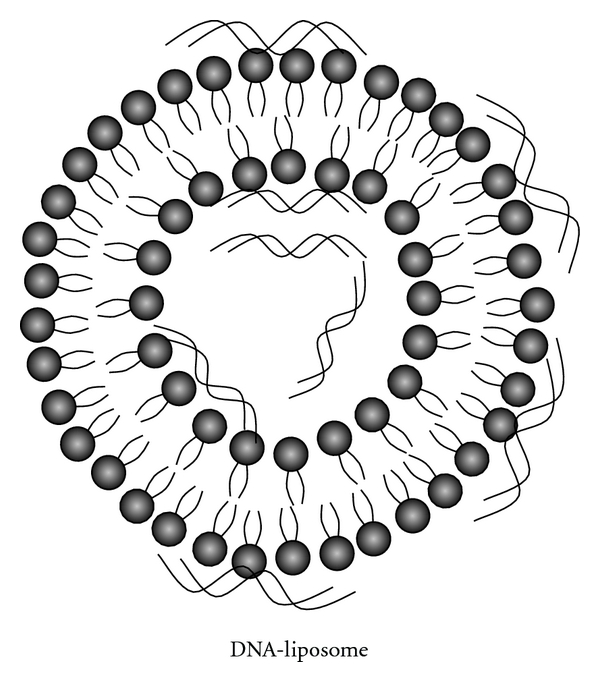
A Schematic representation of a DNA-Liposome complex.

The delivery of the large anionic bioactive DNA across cell has been one of the most difficult endeavours. DNA is easily degraded by circulating and intracellular deoxyribonucleases. Notwithstanding, it must also be delivered intact across the cell and nucleolar membranes to the nucleus. Liposomes have, thus, proved to achieve efficient intracellular delivery of DNA [48]. Such liposomes are prepared from phospholipids with an amine hydrophilic head group. The amines may be either quaternary ammonium, tertiary, secondary, or primary, and the liposomes prepared in this way are commonly referred to as cationic liposomes since they possess a positive surface charge at physiological pH (Figure 4).

Although the experimental data have demonstrated that cationic liposomes can facilitate the transfer of DNA into live mammalian cells, there are still major problems that need to be overcome in order to effectively achieve the goal. These include a reduction in the rapid clearance of cationic liposomes and the production of efficiently targeted liposomes. At the cellular level, the problems may be overcome by improving the receptor-mediated uptake employing appropriate ligands.

## 4. Liposomes as Neuropharmacological Agents

Liposomes are of a great importance as nanocarriers due to their relatively large carrying capacity. They have long been used as drug delivery system to the brain, because the particles can entrap the compounds and prevent the rapid elimination or degradation as well as promote the penetration through the BBB which in turn decreases the effective dose [49]. In addition, they do not elicit negative biological responses that generally occur when a foreign material is introduced in the system. With the pretreatment and adequate formulation in the brain or in places close to the brain, the liposomes are nontoxic, nonimmunogenic, noncarcinogenic, nonthrombogenic, and biodegradable [50].

Based on the same concept the use of liposomes was proposed for the delivery of diagnostic agents across the BBB. For example, it was patented a method using a brain-specific liposome targeting vehicle capable of transporting congo red for neurodiagnostic of Alzheimer's disease, or transporting EGF analogues for brain tumours [51]. Recently, Oku et al. published a new method using PET imaging with positron emitter-labelled liposomes. This method allows accumulating liposomes in brain tumours and then detects small brain tumours with PET scanning [52].

### 4.1. Liposomes in Parkinson's Disease

Parkinson's disease is a progressive neurodegenerative disorder which involves the loss of dopaminergic neurons of the substantia nigra [16, 17, 53]. The neuropathological hallmark of Parkinson's disease is the progressive degeneration of dopaminergic neurons in the substantia nigra pars compacta of the brain in addition to astrocytic gliosis and the presence of numerous other neuronal systems, associated with widespread occurrence of intracytoplasmatic alpha-synuclein positive inclusions known as the Lewy bodies and the Lewy neuritis of neuronal cells [54]. The ubiquitous protein alpha-synuclein is involved in the pathogenesis of Parkinson's disease and comprises protein filaments of ubiquitin and alpha-synuclein that are the primary constituent of Lewy's bodies. Aggregated alpha-synuclein binds the proteasome and potently inhibits proteasomal activity and the dopaminergic neurotransmission [55, 56].

In Parkinson's disease the neurochemical effect is a decline in dopamine concentrations in the basal ganglia. The clinical signs include mass loss and gastrointestinal symptoms such as indigestion and constipation due to the alpha-synuclein pathology in the autonomic nerves and ganglia. Current therapy is essentially symptomatic, and L-DOPA, the direct precursor of dopamine, is still the most effective drug for treating bradykinesia and rigidity associated with the disease. However, during chronic treatment with this drug, after a good initial response, a variety of complications emerge. Currently, the laboratories are working with supplementary agents and antioxidants such as vitamin C and E that act as neuroprotectants [57]. Also there are many investigations ongoing with tissue transplantation of fetal and autologous dopamine containing adrenal medulla and glial cell line neurotrophic releasing factor (GDNF) into the cerebral ventricles or basal ganglia or recently inducing copies of genes into the brain to enhance the production of dopamine. Although this research showed promise for the treatment and cure, new approach is needed to test the efficacy and safety.

The anticholinergic drugs such as biperiden, procycline, orphenadrine, benzhexol, and benztropine are used to improve the tremor and stiffness to a greater degree than akinesia and are overall mildly effective [58]. For this reason, nowadays development of new drugs is increasing and improving pharmacological and pharmacokinetic properties compared with L-DOPA [59].

Practical strategies are, therefore, required to develop a system that can facilitate the transport of new drugs across the BBB for effective management of Parkinsonism. Liposome formulation was developed during the last years as sustained release systems for drugs to the brain, providing more effective transport and increasing L-DOPA concentration in the nigrostriatal system after its chemical and enzymatic degradation [60].

Over 30 years ago, it was developed and characterized a new system with dopamine-containing liposomes which exhibited *in vitro* sustained release of dopamine. These liposomes were stereotactically implanted into the striatum of rats subjected to unilateral lesions of the substantia nigra. This study suggested that dopamine-containing liposomes can partially ameliorate the deficits associated with a rodent model of Parkinson's disease and demonstrate the potential of this technology as a method for the controlled delivery of therapeutic agents into discrete areas of the brain [54].

In 2002 an interesting patent was presented with a method of liposomes containing the pharmacological compound coupled to an antibody-binding fragment which link to a receptor molecule present on vascular endothelial cells of the BBB. This antibody fragment allows to bring and fix the liposome to the wall of the endothelial cells of the BBB and to release the drug just in the receptors of the BBB, allowing the entry of the drug only in the brain. The antibody fragment also has to lack a portion or the entire Fc region of the molecule to minimize clearance of the composition by reticuloendothelial system. The receptors used in this patent were transferrin receptor, insulin receptor, insulin-like growth factor (IGF)-I receptor or IGF-II receptor [61], or glucose transport receptor [62]. Another invention based on the same discovery was presented in 2007; this invention used the liposomes but increasing the mean residence time of a camptothecin compound in the brain tissue and extending the benefit of the drug into the brain [63].

It was also published a promising study that compared neostriatum dopamine concentration after intraperitoneal administration of different drugs for Parkinson's disease and the same drugs in liposomal formulations. This study demonstrated the beneficial formulation of liposomes with better control, delivery and releases in the striatum of the anti-Parkinson agents [64].

Glutathione and its associated enzymes form one of the major antioxidant defense in all cells. There is evidence that nonliposomal glutathione crosses the BBB but with low capacity. Recently, a liposomal preparation of glutathione supplied in a liposomal formulation was indicated as promising therapeutic for neuronal maintenance in Parkinson's disease, autism, and schizophrenia [65].

Newly, several L-DOPA dimeric prodrugs have been encapsulated in unilamellar liposomes of phosphatidylcholine and cholesterol, and administrated intraperitoneal via. This formulation showed about 3 fold increase in basal dopamine levels and a sustained delivery of dopamine in the striatum compared with the treatment of equimolar administration of L-DOPA itself. These experiments demonstrated the better improvement of current drugs only changing the delivery and encapsulation [66]. One interesting example happened with GDNF (glial cell-line-derived neurotrophic factor). However, two open-label trials involving continuous GDNF infusion into the putamen of Parkinson's disease patients were stopped due to lack of good results [67, 68]. Although GDNF did not work properly in these clinical trials GDNF may still be an interesting candidate for the future, the problems described by the trial could be related to dose and mode of delivery of the growth factor, and; therefore, polymer-based drug delivery systems such as liposomes could be valuable in these respects.

Recently, one study showed the clinical application of apomorphine, a dopamine receptor agonist. The main problem of apomorphine is the instability and the need for frequent injections. This group developed apomorphine encapsulated within liposomes to protect it from degradation and enhance the permeability across the BBB. They obtained promising results; the uptake of liposomes into the brain was rapid and prolonged, targeting properly the apomorphine into the damaged brain [69].

### 4.2. Liposomes in Alzheimer's Disease

Alzheimer's disease is the most common form of dementia in the elderly population. The mechanisms underlying this disease are not yet completely clear. Loss of short-term memory, language impairment and disorientation of time are that looking like depression symptoms. At the later stages of the disease, behavioural and psychiatric symptoms develop subsequent to the decline in the motor functions [70]. Genetic and biochemical clues suggest that the progressive production and subsequent accumulation of amyloid-beta plays a role in the Alzheimer's disease pathogenesis. There is no drug to treat Alzheimer's disease completely. Indeed, strategies targeting the production and clearance of amyloid-beta peptide are the most advanced. However, cholinesterase inhibitors such as rivastigmine are the only agents approved by the FDA for treatment of this disease [71].

The objective of the last years is using the current drugs developed with new formulations with nanotechnology. Based on liposome technology, rivastigmine liposomes were developed for delivery into the brain through intranasal route. This study showed that this particular administration with liposomes significantly increased the exposure and the concentration of the drug into the brain [72]. Recently, it was developed a new liposome formulations using DPPC and cholesterol of rivastigmine. This study showed a significantly increasing exposure of the drug into the brain after intraperitoneal and oral administrations compared with drug administration without liposomes [73].

Another example which demonstrates the improvement of the treatment when it is administrated in liposomes was showed with the quercetin. Oral administration of quercetin was able to improve learning and memory ability [74, 75]; however, the main problem is the poor absorption and difficulty to pass the BBB. This problematic was trying to be solved in several papers by Phachonpai et al. in a mouse model of Alzheimer's disease where they demonstrated that nasal administration of Quercetin liposomes attenuated degeneration of cortical neurons and cholinergic neurons in hippocampus [76, 77].

A novel liposome delivery system was also developed for direct transport into olfactory epithelium cells with polyethylene glycol (PEG)ylated immunoliposomes directed against human gliofibrillary acidic protein (GFAP). The handicap of these liposomes is being incapable of penetrating the unimpaired BBB; nevertheless, they may be useful in delivering drugs to glial brain tumours (which continue to express GFAP) or to other pathological loci in the brain with a partially disintegrated BBB such as Alzheimer's disease [78]. Furthermore, this liposome-mediated transport system holds promise for the delivery of bioactive substances to olfactory epithelial cells and modulation of their capacity to stimulate axonal regeneration.

Following the hypothesis that Alzheimer's disease is a conformational disease, the neurotoxic amyloid-beta peptide is formed in anomalous amounts in Alzheimer's disease. This peptide is released as monomer and then undergoes aggregation forming oligomers, fibrils, and plaques in diseased brains. The amyloid-beta aggregates are considered as possible targets for therapy and diagnosis of Alzheimer's disease. Recently it was published a very interesting new potential treatment for Alzheimer's disease, using curcumin that interferes with amyloid plaques encapsulated in liposomes, Mourtas et al. showed an interesting study where they described the binding of curcumin in the fibrils interfering in the new formation of plaques. Although it is a preliminary study, these curcumin liposomes exploit as new vectors to the diagnostic and therapeutic of Alzheimer's disease [79]. another therapy was described by Gobbi et al. where they realized two types of liposomes and solid lipid nanoparticles, 145 and 76 nm average size, respectively. Both liposomes functionalized to target amyloid-beta (1-42) with high affinity. These characteristics make their liposomes a very promising vector for the targeted delivery of potential new diagnostic and therapeutic molecules to be tested in appropriate animal models and clinical trials [80]. Based on the same concept, Canovi et al. characterized the binding properties of nanoliposomes decorated with an anti-amyloid-beta monoclonal antibody obtained in mice by immunization with amyloid-beta antigen followed by hybridoma fusion. When they studied by surface plasmon resonance the liposomes bound to amyloid beta peptides, they showed markedly bound to amyloid-beta monomers and fibrils immobilized on the chip. Interestingly, these liposomes also bound amyloid deposits in postmortem Alzheimer's disease brain samples, confirming the potential of these liposomes for the diagnosis and therapy of Alzheimer's disease [81].

Recent *in vitro* studies with hybrid liposomes suggested new formulations that are able to restore and maintain physiological membrane properties after the toxicity induced by amyloid-beta. In the first study, they investigated the inhibitory effects of hybrid liposomes on the accumulation of amyloid beta 1-40 for SH-SY5Y cells. They prepared liposomes composed by phospholipids having various charged head groups (cationic L-alpha-dimyristoyltrimethyl ammonium propane (DMTAP), anionic L-alpha-dimyristoylphosphatidylserine (DMPS), or zwitterionic L-alpha-dimyristoylphosphatidylcholine (DMPC)) and polyoxyethylene(23) dodecyl ether (C(12)(EO)(23)), and found that the cytotoxicity of amyloid-beta (1-42) peptides on the SH-SY5Y cells decreased after the treatment with this formulation of liposomes [82]. In the other *in vitro* study, they applied unilamellar liposomes in HEK-APP cells, providing protection from oxidation and effective incorporation of omega-3 fatty acid docosahexaenoic acid (DHA) into cell membranes comparing with HEK293 control cells. This study focused in interesting neuroprotection view using liposomes that are able to restore and maintain physiological membrane properties transferring docosanoic acid [83]. These novel studies with new formulation of hybrid liposomes could be used as novel medicine for Alzheimer's disease in the future.

Further, several groups are working in different liposome-based vaccines directed toward different conformations of amyloid beta peptide. Interestingly, incorporation of antigens into biomaterials, such as liposomes, can achieve a desired vaccine response. A promising study demonstrated that liposomal vaccine was more effective when the liposomes carried out antibodies against beta-sheet conformation [84].

## 5. Conclusions

Nanoscale drug delivery systems including liposomes, polymers, and other nanoparticles provide potential solutions to improve neurodegeneration therapeutics. Of these drug delivery systems, liposome-based agents will have the greatest impact in neurology. Current liposomal drugs evolve from a number of design strategies for the improvement in biodistribution over free drugs. Reticuloendothelial system-targeted formulations significantly reduce systemic exposure to high peak levels of free drug but do not facilitate targeting to brain. Passive or physiologic targeting of drugs to brain regions is achievable using long-circulating liposomes, including pure lipid systems as well as surface-modified formulations designed to resist recognition and uptake by reticuloendothelial system cells.

The neurodegeneration of the Alzheimer's disease and Parkinson's disease has not been beneficially treated by classical oral therapy. Levodopa for Parkinson's disease and rivastigmine for Alzheimer's disease remain the gold standard for the therapy. The design and development of an alternative drug based on new technologies will have a key role in the systemic application of new drugs, such as, growth factors, peptides or hormones. Nowadays is impossible to treat correctly many diseases mainly for the localization of damaged tissue or the complexity of tissue affected. The complexity of the disease and, many times, the localization of the tissue damage, difficult the possible treatment, for example, the brain is isolated by the BBB.

It is well demonstrated that the application of neurotrophic factors is able to modulate neuronal survival and synaptic connectivity, and it is a promising therapeutic approach for these neurodegenerative diseases. Although, it is very difficult to ensure long-term administration into the brain, liposome technology allows us to facilitate transport across the BBB. Liposomes have been used clinically as delivery systems for therapeutic drug delivery of chemotherapeutic agents, antibiotics, and antifungals. This is because liposomal preparations have been shown to increase the margin of safety of many drugs and also their efficacy. Among all the applications of liposomal technology, the development of a suitable liposomal carrier to encapsulate neuroactive compounds is very promising. These liposomes are stable enough to be carried to the brain across the BBB, with the appropriate surface characteristics for an effective targeting and for an active membrane transport.

Improvements and adjustments to the liposomal formulation are constantly being explored through the addition of different lipids and targeting molecules. For example, in liposomes lacking cholesterol, high-density lipoprotein can cause disintegration of the liposome, or in liposomes which do contain too much cholesterol, high-density lipoprotein can also cause leakage of contents. The development of novel therapeutic strategies for neurodegenerative and neurological diseases represents one of the biggest unmet medical needs today. The rapid development of liposome technology may provide a nearly solution to overcome these diagnostic and neurotherapeutic challenges for neurodegenerative diseases such as Alzheimer's disease and Parkinson's disease.
